# Volumetric Spatial Correlations of Neurovascular Coupling Studied using Single Pulse Opto-fMRI

**DOI:** 10.1038/srep41583

**Published:** 2017-02-08

**Authors:** Isabel N. Christie, Jack A. Wells, Sergey Kasparov, Alexander V. Gourine, Mark F. Lythgoe

**Affiliations:** 1 UCL Centre for Advanced Biomedical Imaging, Division of Medicine, University College London, London, UK; 2 Centre for Cardiovascular and Metabolic Neuroscience, Neuroscience, Physiology and Pharmacology, University College London, London, UK; 3 Physiology and Pharmacology, University of Bristol, Bristol, UK

## Abstract

Neurovascular coupling describes the link between neuronal activity and cerebral blood flow. This relationship has been the subject of intense scrutiny, with most previous work seeking to understand temporal correlations that describe neurovascular coupling. However, to date, the study of spatial correlations has been limited to two-dimensional mapping of neuronal or vascular derived signals emanating from the brain’s surface, using optical imaging techniques. Here, we investigate spatial correlations of neurovascular coupling in three dimensions, by applying a single 10 ms pulse of light to trigger optogenetic activation of cortical neurons transduced to express channelrhodopsin2, with concurrent fMRI. We estimated the spatial extent of increased neuronal activity using a model that takes into the account the scattering and absorption of blue light in brain tissue together with the relative density of channelrhodopsin2 expression across cortical layers. This method allows precise modulation of the volume of activated tissue in the cerebral cortex with concurrent three-dimensional mapping of functional hyperemia. Single pulse opto-fMRI minimizes adaptation, avoids heating artefacts and enables confined recruitment of the neuronal activity. Using this novel method, we present evidence for direct proportionality of volumetric spatial neurovascular coupling in the cerebral cortex.

The development of functional MRI (fMRI) technology has had the most profound impact on neuroscience and basic cognitive research. Despite its vast application, questions remain about how the blood oxygen level dependent (BOLD) signal relates to neuronal activity. This lack of complete understanding limits our interpretation of the mechanistic origins of the BOLD signal, which are essential to make inferences in basic and clinical neuroscience using this technology. Neurovascular coupling describes the relationship between neuronal activity and resultant changes in local haemodynamics, which underlie the BOLD signal. Accurate characterization of neurovascular coupling is essential for our understanding of the neuronal correlates of fMRI signals.

An experimental approach widely used to study neurovascular coupling is to record correlations between measures of neuronal activity and that of brain hemodynamics. The majority of such correlative analysis has been performed in the temporal domain^1^,^2^,^3^. For example, in the work by Logothetis *et al*.^4^, the temporal profile of the BOLD response to 4, 12 and 24s-long periods of increased neuronal activity, induced by sensory stimulation in awake behaving primates was recorded, with fMRI signals found to most closely correlate with local field potentials. But how do spatial correlations fare? That is, how does modulating the spatial extent of increased neuronal activity affect the spatial extent of the resultant increases of CBF in and around the “active” region? To date, studies of spatial neurovascular correlations have been limited to 2D optical imaging of neural or vascular derived signals that arise from the brain surface^5^,^6^,^7^,^8^. In this study we investigated spatial neurovascular coupling in three dimensions using optogenetic recruitment of cortical neuronal activity in combination with fMRI.

The recent emergence of optogenetic techniques has enabled refined and novel experimentation into brain function^9^,^10^,^11^,^12^,^13^. Distinct cellular populations can be transduced to express light sensitive actuators, allowing their selective excitation/inhibition using light. In this study, conducted in rats, we transduced cortical glutamatergic neurons to express ChR2 and stimulated them using blue light delivered via an optic fibre^10^,^14^,^15^. To investigate spatial correlations of neurovascular coupling, we used a single 10 ms pulse of light of different intensities to modulate the spatial extent of neuronal activation, whilst minimizing both neuronal and vascular adaptations^2^,^16^,^17^. By using such a short stimulus, various light powers can be applied whilst avoiding heating artifacts associated with application of trains of light stimuli of high intensity^18^. Moreover, single pulse stimulation is expected to activate only a limited and local neuronal population^7^,^19^. In this study the extent of optogenetically-induced increases in neuronal activity is estimated (and not measured) using earlier data that describe the scattering and absorption of blue light in brain tissue together with histological assessment of relative ChR2 expression in the cortex. Using this approach, we investigated correlations between the estimated volume of increased neuronal activity and the measured volume of the resultant functional hyperemic response. A key distinction here is the estimation of *volume* rather than *area* of neural and haemodynamic spatial correlations. This distinction is important since estimates of volume represent a closer correlate of the number of “activated” neurons and the degree of additional oxygen and metabolic substrates delivered to the tissue via an increase in CBF. To our knowledge, the methods and results presented here represent the first investigation of spatial neurovascular correlations in three dimensions.

## Results

### Single Pulse Opto-fMRI to Investigate Volumetric Spatial Neurovascular Coupling

**Figure 1:**
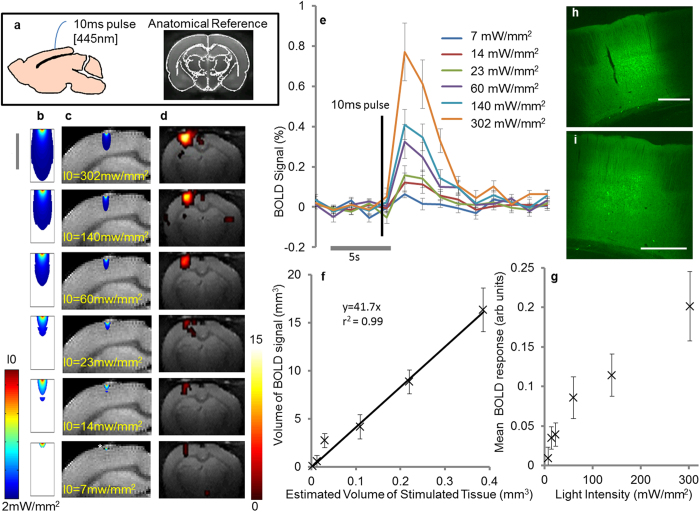
fMRI BOLD Response triggered by Optogenetic Activation of Cortical Neurons Using a Single 10 ms Pulse of Light. (**a**) A single pulse of light (10 ms, 445 nm) was delivered to the rat somatosensory cortex (3 mm caudal to Bregma – anatomical reference is shown) at variable light intensities. (**b**) The volume of stimulated cortical tissue was estimated and the predicted area exceeding threshold intensity for action potential generation is shown within a 2D slice for each of the applied light intensities (I0 = 302, 140. 60, 23, 14, 7 mW mm^−2^ for each row respectively). The scale bar represents 1 mm. (**c**) Schematic illustration of the predicated area of action potential generation in the cortex at variable light intensities (to scale). The predicted volume of stimulated cortical tissue increases with the applied photo energy. * highlights the small indentation in the outer layers of the cortex from the tip of the implanted optic fibre (diameter 200 µm). (**d**) BOLD activation maps induced by a 10 ms pulse of light overlaid on an anatomical reference image at variable light intensities (p = 0.05, uncorrected). The spatial extent of the BOLD response increases with the volume of activated cortical tissue. Data from a single representative experiment are shown. The colour bar represents t scores from 0 to 15. (**e**) The mean BOLD response (+/−1 SEM) across all subjects at different light intensities. For each subject, the BOLD signal is taken from the same voxels. (**f**) The measured volume of BOLD response (taken from the number of “activated voxels”) plotted against the predicted volume of activated neurons receiving light which exceeds the stimulation threshold (2 mW mm^−2^). The mean across the subjects is shown (+/−1 SEM). A linear fit of direct proportionality was applied to the data. (**g**) The mean magnitude of BOLD response (see panel D) 8 seconds following light delivery plotted against light intensity. The mean across all subjects is shown (+/−1 SEM). (**h/i**) Representative images of coronal sections of the rat brain illustrating the extent of cortical ChR2 expression in cortical neurons following microinjections of AAV2-CamKIIa- hChR2(H134R)-eYFP. Scale bar = 500 μm.

In alpha-chloralose (75 mg kg^−1^, IV) anaesthetised rats, robust localized BOLD responses were detected following optogenetic activation of cortical ChR2-expressing neurons triggered by a single 10 ms pulse of light (Fig. 1). Light intensity was gradually increased to modulate the spatial extent of the stimulus (Fig. 1a–c). The estimated volume of cortical neurons exceeding the intensity threshold required for action potential generation (2 mW mm^−2^) was found to be dependent on the applied light power (Fig. 1b,c). With the increasing light intensity both the magnitude (Fig. 1e,g) and spatial extent (Fig. 1d,f) of the recorded BOLD signal increased. The time-course of the BOLD response triggered by a single 10 ms pulse of light shared some characteristics with those previously reported to be evoked by short sensory stimulations (Fig. 1e). Following application of a single 10 ms light pulse there was a delay (~1 s) prior to a rapid increase in BOLD signal that returned to baseline within the following 8–10 s, similar to that previously reported in some event related studies (e.g. refs 20,21).

Data presented in Fig. 1(f) confirms that the spatial extent of the BOLD response exceeds the estimated volume of neuronal activation, as previously reported^22^,^23^. Despite this spatial miss-match, a directly proportional relationship was observed between the volume of the evoked BOLD response and the estimated volume of ‘light activated’ brain tissue occupied by ChR2 expressing cortical neurons (Fig. 1(f)- the individual subject data are shown in Supplementary Figure 1). Robust direct-proportionality of spatial neurovascular coupling was observed across the whole range of examined ‘average activation thresholds’ (1–3 mW mm^−2^ –Supplementary Figure 2). No consistent activations in remote brain regions were observed in these experiments. Histological examination of the efficacy of viral transfection of cortical neurons confirmed that the extent of ChR2 expression exceeded the estimated volume of activated neurons (Fig. 1h,i).

### BOLD fMRI Responses Triggered by a Single Pulse of Light: No Evidence of Adaptation

**Figure 2:**
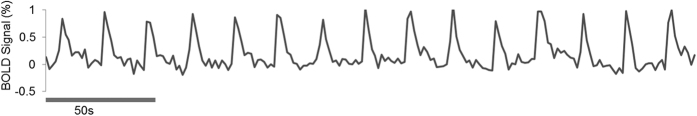
The BOLD Response Triggered by Optogenetic Activation of Cortical Neurons by Application of a Single Pulse of Light Displays no Evidence of Adaptation. The mean BOLD fMRI signal (n = 7, continuous, raw data) to 15 consecutive pulses of light (302 mW mm^−2^, 10 ms pulse width, ISI = 20 s).

Based on the existing evidence^2^,^12^,^16^,^17^, we reasoned that a single 10 ms pulse of light (inter-stimulus-interval (ISI) = 20 s) would not lead to vascular adaptation (in contrast to a repeated stimulation). Figure 2 illustrates the BOLD fMRI responses to 15 repeated single-pulse stimuli acquired in sequence (averaged over all the subjects when the light was applied at the greatest intensity [302 mW mm^−2^]). No trend for diminishing BOLD response was observed with repeated optogenetic activations. Hence adaption does not appear to confound neurovascular coupling experiments in the single pulse experimental paradigm.

### A Single Pulse Stimulation Produces Negligible Heating-Related ‘Pseudo-fMRI’ Responses

**Figure 3:**
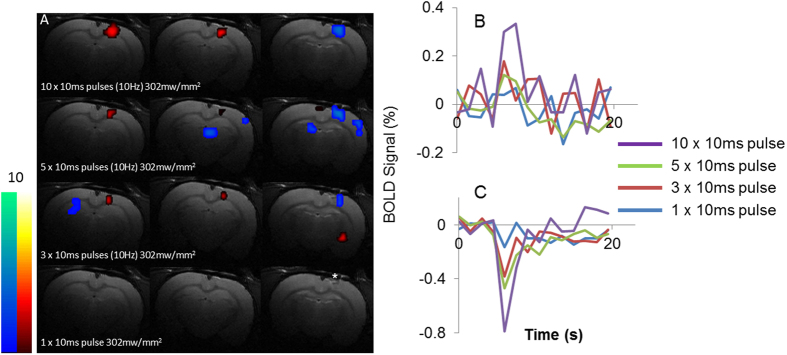
Pseudo BOLD fMRI Responses triggered in the Naïve Rat Brain. (**A**) Cortical BOLD “pseudo-activation” maps from a naïve rat (no expression of optogenetic proteins) overlaid on an anatomical reference scans. Maps of “pseudo-activation” are shown following a single 10 ms pulse (the same protocol as applied in the main experiment; Fig. 1) as well as 3, 5 and 10 pulses at 10 Hz at a light intensity of 302 mW mm^−2^. The point of light delivery is indicated by *. ‘Positive’ and ‘Negative’ activations are observed in adjacent coronal slices as previous described^18^. The colour bar represents t scores from 0 to 10. (**B**) The mean positive “pseudo-BOLD” responses evoked by a different number of light pulses at the point of light delivery. The positive “pseudo-BOLD” signal is taken from the same positive voxels, “activated” with 10 pulses. (**C**) The mean negative “pseudo-BOLD” response evoked by a different number of light pulses at the point of light delivery. The negative “pseudo-BOLD” signal is taken from the same negative voxels, “activated” with 10 pulses.

A single pulse light stimulation paradigm was applied in three naïve rats (no ChR2 expression), to investigate the extent of possible ‘pseudo-fMRI’ signals associated with brain tissue heating^18^. Figure 3 shows fMRI ‘pseudo-activation’ maps and fMRI time-series data following a single pulse simulation and following application of 3, 5 and 10 repeated light pulses respectively (all at the greatest light intensity applied in the experiments described above [302 mW mm^−2^]). Characteristic positive and negative fMRI signals were observed in adjacent coronal slices at the point of light delivery following 10 repeated pulses in agreement with previous observations^18^. The extent of pseudo-fMRI signals decreased with lowering the number of repeated pulses of light as expected. A single pulse produced negligible fMRI signal changes at the greatest applied light intensity in comparison to that triggered in rats transfected to express ChR2 in cortical neurons (Fig. 1). Therefore, ‘pseudo-fMRI’ signals related to tissue heating do not confound fMRI BOLD responses illustrated in Figs 1 and 2. Some ‘positive’ and negative ‘blobs’ were observed at a distance from the site of light delivery but these were not consistent across the different number of repeated light pulses and likely to represent false positives due to a relatively low detection threshold (p < 0.05, uncorrected).

## Discussion

Despite extensive characterization of temporal correlations that describe neurovascular coupling, the study of spatial correlations has so far been confined to 2D imaging of signals emanating from the surface of the brain^7^,^22^,^24^. Thus, it remains unknown, for example, how changes in the volume occupied by activated neurons change CBF within and around the activated brain region. In this study, we developed and applied a new experimental paradigm involving optogenetic activation of cortical neurons using a single pulse of light. We modulated the volume of neuronal excitation in a controlled manner by delivering a single 10 ms pulse of blue light of variable intensity directly to the rat cortex transduced to express ChR2. This approach provides a unique perspective on the spatial dynamics of neurovascular coupling, without the confounds of adaptation and heating associated with the delivery of multiple pulses involving prolonged stimulation paradigms, and the methodological confines imposed by the discrete point source nature of electrophysiological recording devices. This approach exploits the compatibility of fibre optics and MRI, and avoids the susceptibility artifacts and electromagnetic interference introduced by the recording electrodes.

Previous studies have shown that, though well co-localized^24^,^25^, the spatial extent of the haemodynamic response appears to markedly exceed the volume of heightened neuronal activity^7^. The reasons for this phenomenon (often likened to “watering the garden for the sake of a flower”)^22^, are not yet known^23^. Given this phenomenon, it is difficult to accurately predict the spatial extent of neuronal activation from the recorded BOLD response. Here, we present evidence for direct proportionality between the estimated volume of increased neuronal activity and the spatial extent of the BOLD fMRI response, triggered by application of a single pulse of light (Fig. 1,e). Despite the well-recognized spatial mismatch between neuronal activity and the BOLD response, these findings suggest that the cortex scales the spatial extent of the hemodynamic response in direct proportion to the volume of increased neuronal activity. Thus, under these experimental conditions and across the ranges of spatial scales tested here, the spatial extent of BOLD responses in the cortex is a linear multiplying function determined by the volume occupied by activated neurons.

This investigation parallels another study that used optical imaging spectroscopy to investigate the spatial extent of the haemodynamic response to optogenetic stimulation in transgenic mice expressing ChR2 in layer 5 pyramidal neurons^7^. The authors estimated the area of ChR2 activation (based on the ChR2-YFP fluorescent signal) and light-induced changes in neuronal activity (via measures of local field potentials and multi-unit activity). In doing so, the authors estimated the point spread function of the haemodynamic response surrounding the area of increased neuronal activity. The point spread function is of fundamental importance to the maximum spatial resolution that fMRI signals (as a surrogate marker of changes in neuronal activity) can theoretically achieve. The authors reported that the spatial extent of the neuronal responses was confined to the region of ChR2 expression within ~250–350 μm. However, in this previous study, no discernible trend between the intensity of the applied stimulus and the spatial extent of neuronal activation was found in response to a single pulse of light. However in this study^7^ expression of ChR2 was restricted to layer 5 by virtue of the transgenic animal model. In our study, ChR2 expression in neurons of all cortical layers was induced by viral vectors using a CaMKII promoter. In the context of ultra-resolution fMRI^26^ in which laminar-specific BOLD responses can be resolved, the impact of optogenetic stimulation of multiple cortical layers warrants discussion. The majority of pyramidal neurons are spread between layers 4 and 6^27^,^28^, but layer 5 is the main site of action potential generation in response to sensory stimulation^29^. The way in which optogenetic stimulation of cortical neurons was applied in this study, is different to sensory stimulation, in which neurons of layer 4 are the principal recipients of thalamocortic afferent inputs^29^. However a discordance between the peak of multiunit activity (layer 5) and the peak of CBV responses (layer 4) has been reported^26^, suggesting that hemodynamic responses may not only reflect local neuronal activity, but may be contaminated by the proximity to microvasculature^26^. Therefore, for the purposes of this study (understanding the volumetric spatial correlation between activated brain tissue and the BOLD response), transducing neurons of all cortical layers to express optogenetic actuators seemed most appropriate.

One important limitation of the current study is that the spatial extent of neuronal activation was estimated and not directly measured. We used a single pulse stimulation paradigm to maximize the accuracy of these estimates as a single pulse (i) avoids adaptation (Fig. 2), (ii) induces no heating artefacts even at high light intensities that are required to recruit a large volume of neurons (Fig. 3) and, (iii) results in relatively confined neuronal recruitment (relative to longer trains of stimuli)^7^,^19^. Whilst it would have been preferable to perform 3D mapping of neuronal activity changes in an analogous way to fMRI BOLD changes, we are currently limited by the available methods (since electrophysiological recording devices are essentially point source recorders and optical imaging of voltage sensitive dyes captures 2D surface data). In this study, the volume of light-activated cortical neurons was estimated based on the well characterized scattering and absorption properties of blue light in brain tissue^10^,^14^,^15^, together with layer specific assessment of the density of ChR2 expression (taken from the relative YFP expression (from the histology))^30^. The absolute ChR2 expression density is unknown and therefore we tested several ‘average activation thresholds’ (1–3 mW mm^−2^) weighted by the estimates of relative ChR2 expression density across all cortical layers. Supplementary Figure 2 illustrates direct proportionality of volumetric neurovascular coupling which is preserved across all tested activation thresholds. This relatively simple model does not account for the cytoachitecture of the cortex or the distribution of ChR2 expression at cellular level (i.e. the relative distribution of ChR2 expression across the cell soma vs processes for example). Nonetheless, the data reported provides clear evidence for a direct proportionality of volumetric spatial neurovascular coupling in the rat cortex (not necessarily an obvious outcome given the marked spatial miss-match inherent to neurovascular coupling). Future studies may be designed to employ a more advanced experimental models that take into the account quantitative cortical ChR2 expression and its cellular distribution. The observed direct proportionality of volumetric spatial neurovascular coupling may be dependent on the short stimuli used (10 ms). Indeed, an earlier study observed direct proportionality of temporal neurovascular coupling with short stimuli, which disassociated when longer trains of repeated stimuli were applied^2^. It would be interesting to have extended the study of volumetric spatial correlations over a greater range of stimulus durations, however this may have violate points i-iii) (described above) that form the basis of our study design to maximize the accuracy of the estimated volume of optogenetically induced action potentials by using single-pulse stimulation. In this study we achieved extensive cortical expression of ChR2 (see Fig. 1h,i) which ensured that the estimated volume of stimulated neurons is not limited by the extent of transgene expression.

In conclusion, in this study we combined optogenetics and fMRI to investigate volumetric spatial correlations of neurovascular coupling. Despite the spatial extent of the recorded BOLD response markedly exceeding the estimated volume occupied by activated neurons, we observed a directly proportional relationship between the predicted volume of optogenetically-induced action potential firing and the spatial extent of the recorded BOLD fMRI response. These data indicate that BOLD fMRI mapping provides a linear spatial correlate to the underlying volume of brain tissue occupied by activated neurons.

## Methods

The experiments were performed on Sprague-Dawley rats in accordance with the European Commission Directive 86/609/EEC (European Convention for the Protection of Vertebrate Animals used for Experimental and Other Scientific Purposes) and the UK Home Office (Scientific Procedures) Act (1986). All experiments were approved and licensed by the UK Home Office.

### Stereotaxic delivery of viral vectors and cortical cannula implantation

Adult male rats (n = 8) were anaesthetised with a mixture of ketamine 60 mg kg^−1^ and meditomidine 250 μg kg^−1^ i.m. The head was positioned in a stereotaxic frame and protective ophthalmic gel was applied. A midline incision was made and a craniotomy (~1 mm^2^) was performed on the right parietal bone. Glass micropipettes containing viral particle suspensions were lowered into the cortical parenchyma of the somatosensory hind paw region of the cortex^31^. Positive pressure was applied to inject the viral suspension (AAV2-CamKIIa-hChR2(H134R)-eYFP; UNC Vector Core, diluted 1:1 with saline) of 1 μl per site (~0.2 μl min^−1^) at six coordinates between A.P. −2.0:−3.5; M.L. + 3.5: + 2.0; D.V. −1.5:−1 mm from Bregma. The pipette was removed 2 minutes after the last injection was completed. Then the guide cannula was implanted into the area of transduced cortex as described in detail previously^18^. The skin was sutured around the cannula, buprenorphine was administered (0.05 mg kg^−1^, s.c.) and anesthesia was reversed with atipamezole (1 mg kg^−1^, i.m.). Post-operative care was given and animals’ appearance and body weight were monitored during the recovery period.

### Surgical preparation for fMRI

fMRI experiments were conducted 21 ± 2 days after viral injections to ensure high and stable level of transgene expression. Anesthesia was induced with isoflurane (5% in air) and the femoral artery and vein were cannulated for measurement of the arterial blood pressure and administration of drugs, respectively. Alpha chloralose was administered (75 mg kg^−1^ i.v.) and isoflurane was discontinued. The trachea was cannulated and the animal was transferred to the MRI scanner bed. The head was secured with tooth and ear bars and the animal was ventilated with oxygen enriched gas mixture using an MRI compatible ventilator (CWE Inc) with a tidal volume of ~1 ml 100 g^−1^ of body weight and a ventilator frequency similar to the resting respiratory rate (~65 breaths min^−1^). During imaging the animal was administered with gallamine triethiodide (50 mg kg^−1^, i.v.; then 10 mg kg^−1^ h^−1^, i.v.). *P*O_2_, *P*CO_2_ and pH of the arterial blood were measured regularly and kept within the physiological ranges by altering tidal volume and ventilator frequency. The body temperature was maintained at 37.0 ± 0.5 °C.

### fMRI

Images were acquired using a 9.4 T Agilent horizontal bore scanner with a single loop surface coil for signal transmission and reception (ImGenius, London, UK).

The tip of the optic fibre (diameter 200 μm) was inserted ~200 μm into the cortical parenchyma (see Fig. 1, Supplementary Figure 3). A laser (Omicron Lasers, Germany) synced to the MRI scanner using a data logger (Cambridge Electronic Designs, UK) was used to deliver blue light (445 nm). A high resolution anatomical reference scan was acquired with the following parameters: TR/TE_eff_ = 3100/48 ms, ETL = 8, matrix size = 256 × 256, FOV = 35 mm × 35 mm, 30 slices, 1 mm slice thickness. Functional images were centered around the point of light delivery (3 mm caudal to Bregma) and were acquired using single shot gradient echo EPI sequence with the following parameters: TR/TE = 1500/15 ms, matrix size = 64 × 64, FOV = 35 mm × 35 mm, 8 slices, 1 mm slice thickness. For the experiments with cortical stimulation using single pulse of light (n = 8), light was delivered for 10 ms with an inter-stimulus interval (ISI) of 20 s, repeated 15 times giving a total acquisition time of approximately 5 min. Data were acquired at variable light intensities (I0 = 7, 14, 23, 60, 140 and 302 mW mm^−2^) and each fMRI sequence was repeated twice at each light intensity in an interleaved manner. The fMRI timecourse data from the repeated scans were averaged together for the subsequent analysis. The power of light emitted by the optic fibre was measured using a power meter (Omicron, Germany).

The same paradigm at the greatest light intensity (302 mW mm^−2^) was also applied to three naïve rats (no optogenetic proteins expressed in the cortex) to determine the extent of previously described^18^ heating-related pseudo-fMRI responses under these specific experimental conditions. In addition, data were also acquired at the greatest applied light intensity (302 mW mm^−2^) with trains of 3, 5 and 10 single pulses (10 Hz, ISI = 20 s) as a positive control in order to confirm delivery of the light to the brain in these animals.

### fMRI data Analysis

For all functional scans, activation maps were generated at each applied light intensity from the GE-EPI data using SPM8 (http://www.fil.ion.ucl.ac.uk/spm/). The GE-EPI images were not spatially smoothed (as is common to fMRI analysis) in order not to decrease the spatial affinity of the estimated volume of BOLD response to the volume of increased CBF. First-level analysis of each subject’s time series using an on/off regressor derived from the applied light delivery paradigm (and convolved with a standard HRF) was applied. A threshold of p < 0.05 (cluster size = 3) was used to generate activation maps illustrating results of the experiments involving application of a single pulse of light. Although this was not corrected for multiple comparisons, activation was observed at the point of light delivery (where the optic fiber penetrates the cortex) at most light intensities tested in all subjects (see Fig. 1) indicating a genuine BOLD response and not false positive data. The cluster size of 3 was chosen as a compromise between permitting the detection of BOLD changes across a small volume of tissue and reducing false positive activation that would otherwise reduce the accuracy of the BOLD volume estimates. The number of “activated” voxels registered at each light intensity was then taken within the volume of activation at the greatest light intensity within the cortical hemisphere where the optic fibre was positioned. In one of the animals, the estimates of the number of activated voxels fell outside 2 standard deviations of the mean at each of the 6 light intensities tested, therefore, these data were excluded from the subsequent analysis. The individual animal estimates of the spatial extent of the BOLD response, at each light intensity, are reported in the Supplementary Material. A fit of direct proportionality (y = mx) was performed on the predicted volume of neural tissue exceeding action potential generation (see below) against the mean volume of BOLD activation (estimated based on the number of activated voxels with a size of ~0.3 mm^3^). To estimate the volume of activated tissue by a single 10 ms pulse of light delivered at variable intensities, we used a model described by Foutz *et al*., (ref. 15 [Eqs 11–20]) that describes the scattering and absorption of blue light in brain tissue (that is, the relative intensity of the light as a function of distance from the optic fibre). We estimated the extent of ChR2 expression across the cortical layers by measuring the florescent signal (GFP fluorescence) across the cortex (described below). The mean florescent signal was taken across each of the cortical layers and the relative intensity (arbitrary units) was found to be the following: (layer 2/3 = 0.71; layer 4 = 1.08; layer 5a = 1.24; layer 5b = 1.24; layer 6 = 0.93). The depth of the cortical layers was taken from previous work^32^. The relative mean fluorescent signal was taken as an estimate of relative density of ChR2 expression across the cortical layers. Assuming a linear relationship between the estimated ChR2 density and the light ‘activation-threshold’ (an approximation for the relationship^18^, we estimated the volume of ‘light-activated’ tissue to be the volume of tissue in which the light intensity exceeded the layer specific ‘activation threshold’ (calculated for each layer by weighting an ‘average activation threshold’ by the relative ChR2 density across the cortical layers). For example, if the ‘average activation threshold’ was 2 mW mm^−2^ then the layer specific ‘activation threshold’ for layers 2/3 would be 2.8 mW mm^−2^ (2/0.71) and for layer 5 A would be 1.6 mW mm^−2^ (2/1.24). An ‘average activation threshold’ of 2 mW mm^−2^ was selected based on the work of Ishizuka *et al*.^30^, which describes the light intensity required to induce an action potential in isolated pyramidal neurons. However, given that the precise ‘activation threshold’ was unknown, the relationship between the estimated volume of light-activated tissue and the measured size of the BOLD response was also examined using alternative fixed’ average activation thresholds’ of 1, 1.5, 2.5, and 3 mW mm^−2^ (results reported in Supplementary Figure 2).

To estimate the magnitude of BOLD response at the point of light delivery (single 10 ms pulse), the raw fMRI time series data were taken within the “activated” voxels at the minimum light intensity that returned “activated” voxels thresholded at p = 0.05 with FWE correction. For each subject, the same region was used for all light intensities.

### Histology

At the end of all the experiments, the rats were transcardially perfused with normal saline (0.9% NaCl), followed by ice cold paraformaldehyde (PFA, 4%). Brains were post-fixed in PFA for 24 hours, then cryoprotected in sucrose (30%) for 24 hours and serially sectioned (40 μM) using a freezing microtome and slices were mounted on slides. DAPI mounting medium was applied (Vectorshield, Vector Laboratories) and images were acquired using a fluorescent microscope (Zeiss Imager Z.1, Zeiss, Germany). Expression of hChR2 by neurons in the somatosensory cortex was confirmed by the distribution of eYFP fluorescence, as described in previous publications^33^. The intensity of fluorescent signal across the cortex was determined and the mean signal intensity across all cortical layers was calculated (using estimates of cortical layer thickness from previous work)^32^. The mean signal was taken as an estimate of relative density of ChR2 expression to inform estimates of the volume of ‘light-activated’ tissue (see above).

## Additional Information

**How to cite this article**: Christie, I. N. *et al*. Volumetric Spatial Correlations of Neurovascular Coupling Studied using Single Pulse Opto-fMRI. *Sci. Rep.* **7**, 41583; doi: 10.1038/srep41583 (2017).

**Publisher's note:** Springer Nature remains neutral with regard to jurisdictional claims in published maps and institutional affiliations.

## Supplementary Material

Supplementary Figures
