# Supplementary material for: Volumetric Spatial Correlations of Neurovascular Coupling Studied using Single Pulse Opto-fMRI
Source: Sci Rep. 2017 Feb 8;7:41583. doi: 10.1038/srep41583 (PMC5296864; doi:10.1038/srep41583)
Supplement: Supplementary Figures [file srep41583-s1.pdf]

<u>Supplementary Figures:</u> Volumetric Spatial Correlations of Neurovascular Coupling Studied using Single Pulse Opto-fMRI

Isabel N Christie*, Jack A Wells*, Sergey Kasparov, Alexander V Gourine, Mark F Lythgoe

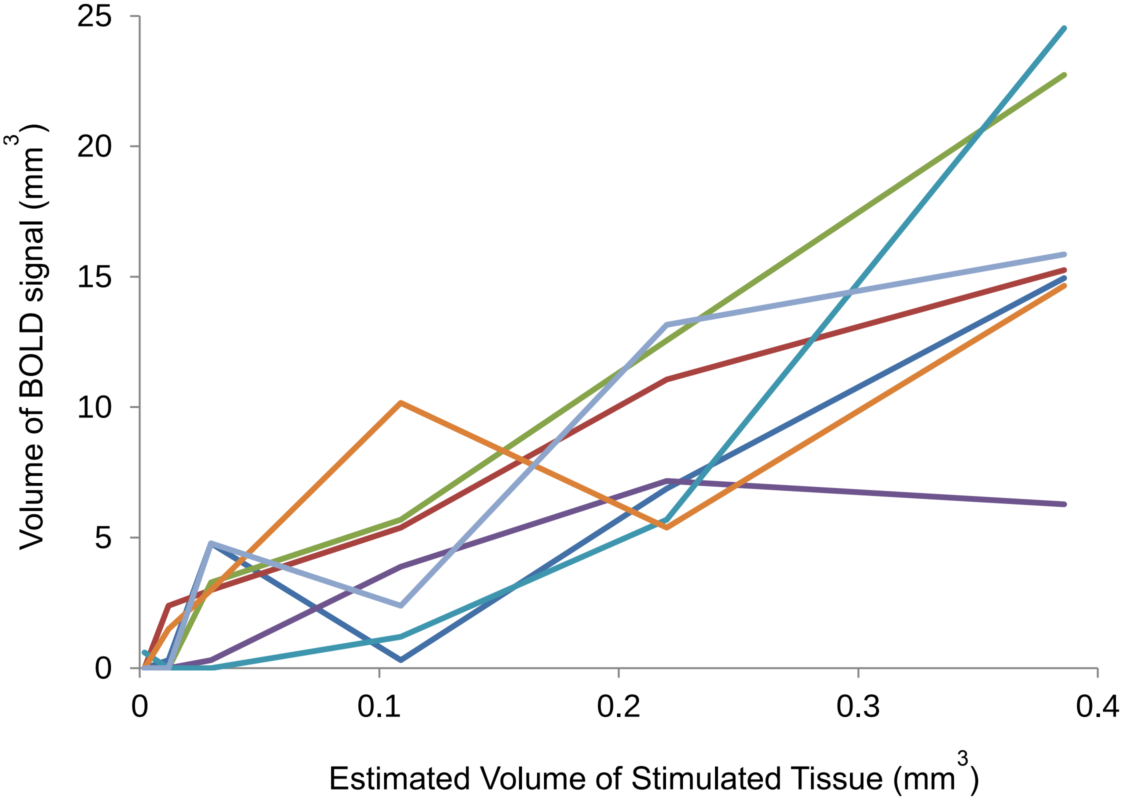

Supplementary Figure 1 : Individual Subject Spatial Neurovascular Coupling (10ms pulse)

The spatial extent of the BOLD response (the number of “activated” voxels) against the predicted volume of action potential generation for each individual subject ('Average Activation Threshold' =2mW/mm^2). The mean value across all subjects is reported in Figure 1 F.

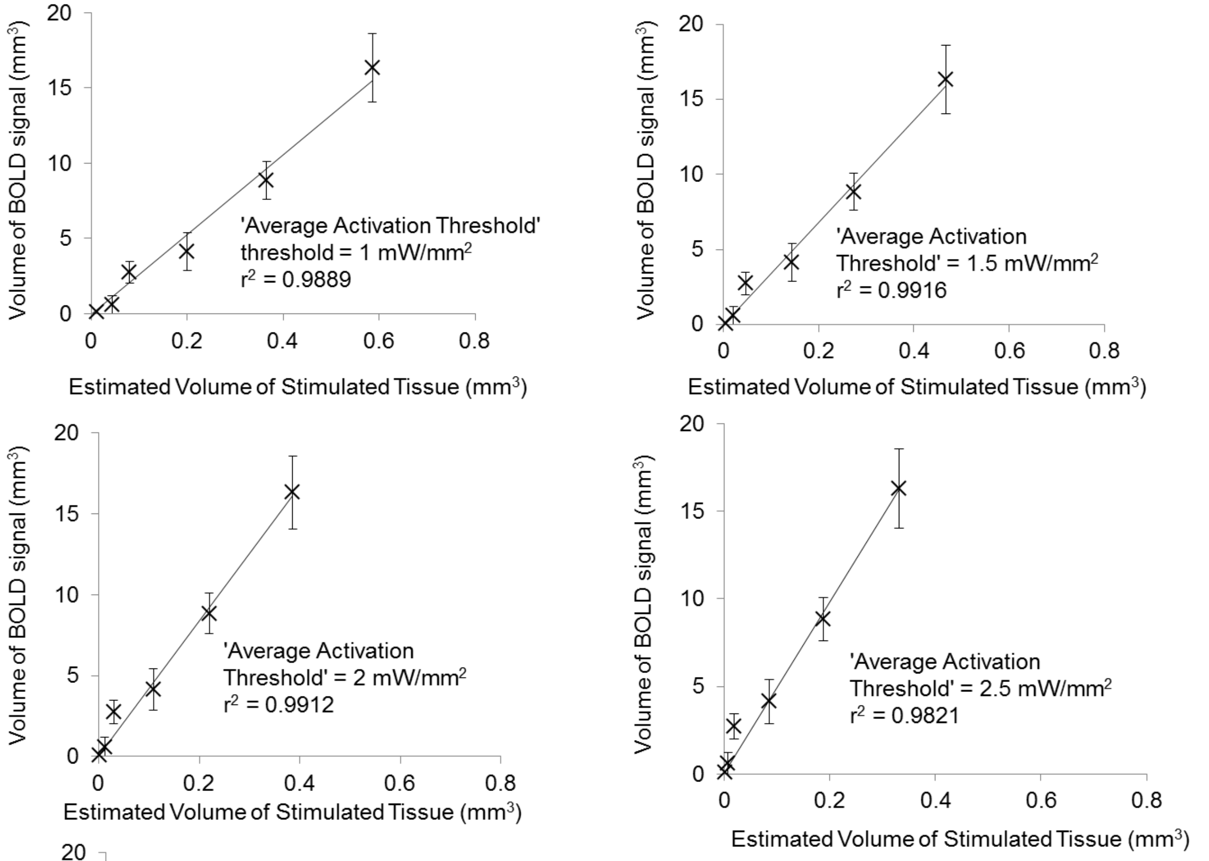

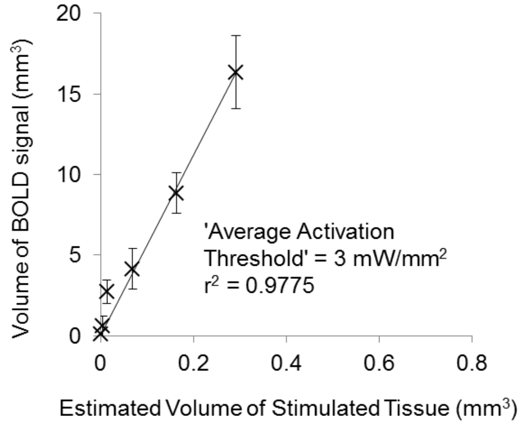

Supplementary Figure 2: Direct Proportionality of Spatial Neurovascular Coupling (single pulse) is Robustly Maintained Across a Range of 'Average Activation Thresholds'

The measured volume of BOLD response is plotted against the estimated volume of stimulated tissue for a range of 'average action potential thresholds (1 – 3 mW/mm^2)'. In each case, linear fits of direct proportionality are displayed.

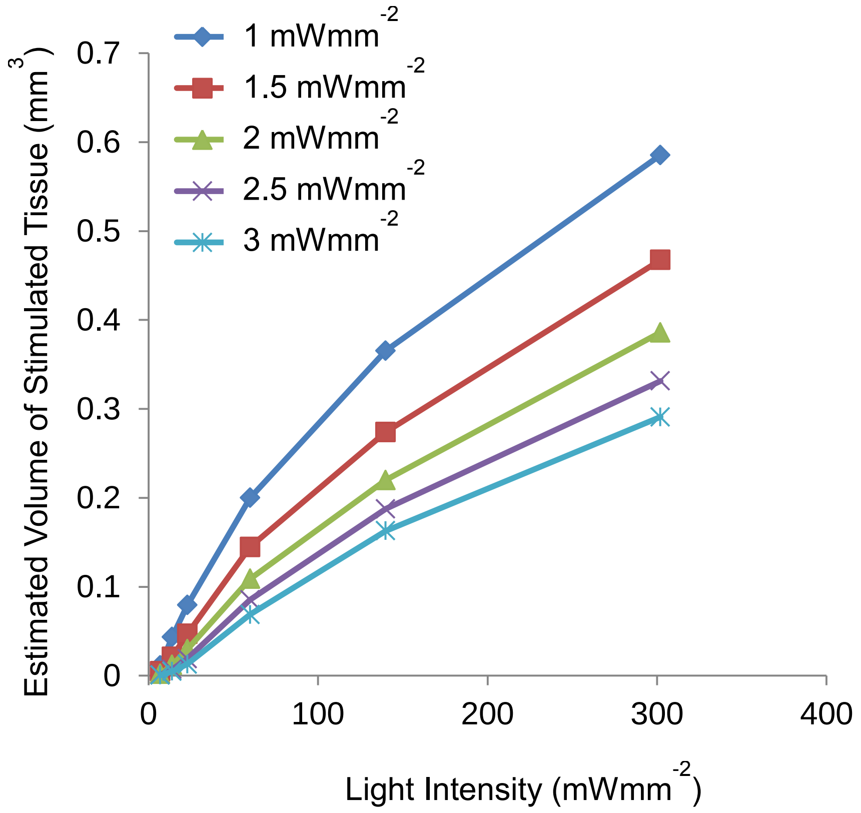

Supplementary Figure 3: Modelled Spatial Extent of Neuronal 'Activation' at Variable Light Intensity

The estimated volume of 'activated' tissue against the delivered light intensity at 'average activation thresholds' between 1 and 3 mW/mm^2.
